# Crowdsourcing taste research: genetic and phenotypic predictors of bitter taste perception as a model

**DOI:** 10.3389/fnint.2014.00033

**Published:** 2014-05-27

**Authors:** Nicole L. Garneau, Tiffany M. Nuessle, Meghan M. Sloan, Stephanie A. Santorico, Bridget C. Coughlin, John E. Hayes

**Affiliations:** ^1^Genetics of Taste Lab, Department of Health Sciences, Denver Museum of Nature and ScienceDenver, CO, USA; ^2^Department of Mathematical and Statistical Sciences, University of Colorado DenverDenver, CO, USA; ^3^Sensory Evaluation Center, Pennsylvania State University, University ParkPA, USA; ^4^Department of Food Science, College of Agricultural Sciences, Pennsylvania State UniversityUniversity Park, PA, USA

**Keywords:** genetics, taste, citizen science, crowdsourcing, *TAS2R38*

## Abstract

Understanding the influence of taste perception on food choice has captured the interest of academics, industry, and the general public, the latter as evidenced by the extent of popular media coverage and use of the term supertaster. Supertasters are highly sensitive to the bitter tastant propylthiouracil (PROP) and its chemical relative phenylthiocarbamide. The well-researched differences in taste sensitivity to these bitter chemicals are partially controlled by variation in the *TAS2R38* gene; however, this variation alone does not explain the supertaster phenomenon. It has been suggested that density of papillae, which house taste buds, may explain supertasting. To address the unresolved role of papillae, we used crowdsourcing in the museum-based Genetics of Taste Lab. This community lab is uniquely situated to attract both a large population of human subjects and host a team of citizen scientists to research population-based questions about human genetics, taste, and health. Using this model, we find that PROP bitterness is not in any way predicted by papillae density. This result holds within the whole sample, when divided into major diplotypes, and when correcting for age, sex, and genotype. Furthermore, it holds when dividing participants into oft-used taster status groups. These data argue against the use of papillae density in predicting taste sensitivity and caution against imprecise use of the term supertaster. Furthermore, it supports a growing volume of evidence that sets the stage for hypergeusia, a reconceptualization of heightened oral sensitivity that is not based solely on PROP or papillae density. Finally, our model demonstrates how community-based research can serve as a unique venue for both study participation and citizen science that makes scientific research accessible and relevant to people’s everyday lives.

## INTRODUCTION

Taste sensitivity is relevant to our everyday lives. It is then no surprise that the role of taste variation in health has captured the interest of industry, health professionals, and the public alike. Substantial interest in recent years has centered on the term supertaster. It is paradoxically a broad superlative and a narrowly defined phenotype, complicating its general use within the public lexicon. This confusion is furthered due to its popular and continued use in the media and popular science communications to describe anyone with a sensitive palate (e.g., Weir, 2010; Tepper and Keller, 2011; Can I Eat That, 2012; Reddy, 2013; The Numbers, 2013; Your Brain A User’s Guide: 100 Things You Never Knew, 2013). Despite this inaccurate usage, the precise definition is much narrower; supertasters are defined as a subgroup of individuals who report intense bitterness specifically from the chemical propylthiouracil (PROP) and phenylthiocarbamide (PTC; Bartoshuk, 1991; Hayes et al., 2008).

The most well-researched taste phenotype, the variable bitterness of PROP is largely due to three single nucleotide polymorphisms (SNPs) in the gene *TAS2R38* (Kim et al., 2003). These SNPs are responsible for consistently observed bimodal detection thresholds – statistical estimates of the lowest concentration that can be sensed – for PROP and PTC across the population (Blakeslee, 1932; Fox, 1932; Hayes et al., 2008). Subsequent work noted that those with low detection thresholds (“tasters”) could be further divided, as there exists a subgroup of tasters who report intense bitterness from suprathreshold concentrations of PROP; the term supertaster was coined to refer to these individuals (Bartoshuk, 1991). In a small but seminal study, PROP supertasting was found to be correlated to a high density of fungiform papillae (FP) on the tongue, as well as other oral sensations like sweetness and capsaicin burn (Bartoshuk et al., 1994). Subsequently, the term supertaster became synonymous with elevated taste intensity, elevated oral somatosensation, and high FP density (Hayes and Keast, 2011). Both the term and this latter characteristic of supertaster grew in popularity and have become widely accepted by both the media and, consequently, the general public. Meanwhile, due to advances in methodology and technology, scientists have begun to reexamine the defining characteristics of a supertaster (Reed, 2008; Hayes and Keast, 2011) and the positive correlation of FP density to PROP sensitivity is no longer uniformly accepted in the field (Delwiche et al., 2001; Yackinous and Guinard, 2001; Fischer et al., 2013). This shift has not garnered the same media attention and there is now a discord between current scientific understanding and the public perception of the phenomenon.

In light of this, the Denver Museum of Nature & Science (Museum) felt both a need and a unique means to address this discordance through public participation in research. One of the historical challenges of human behavioral research on taste has been small sample sizes, coupled with labor-intensive data processing which often leads to conflicting findings such as the FP density-supertasting correlation. The Museum’s permanent health exhibit, Expedition Health, is open 364 days a year and sees over 400,000 visitors during that time; our access to a large cohort of human participants combined with our trained core of volunteers allowed us to conduct research using models of crowdsourcing and citizen science. Executed correctly, these models can advance scientific discovery while concurrently providing a source of awareness and engagement for a general audience via authentic and active research (Silvertown, 2009; Hand, 2010). By implementing these models, the Lab had the unique resources to ask and answer the unresolved question in taste research: does the density of FP on one’s tongue predict the perception of bitterness of the chemical tastant PROP in the phenomenon known as supertasting?

## MATERIALS AND METHODS

### PARTICIPANTS

Participants in the “Bitter Study” hosted in the Genetics of Taste Lab are Museum guests who elect to enhance their visitor experience by participating in an authentic human genetics research study in the context of taste and health. Our study sample consists of 394 healthy, non-smoking participants who participated in every data collection station and subsequently provide full data sets of the required variables of age, sex, PROP intensity score, FP density, and *TAS2R38* diplotype. All procedures were approved by the Western Institutional Review Board. Written consent was obtained and participants volunteered their time.

#### Citizen science model

Volunteer citizen scientists underwent a 12 week certification program. The program includes detailed trainings on internal quality control for data collection, an online ethics course for working with human populations^1^, and visitor experience instructional sessions for educational facilitation. The program concludes with a final certification, and successful citizen scientists then received approval to enroll visitors over the age of 18 in this research study. Certified citizen scientists also had the opportunity to become trained in data processing and data analysis protocols. They were trained to extract and purify DNA, to prepare and analyze the gene sequencing reactions, and to count and analyze highly labor intensive FP density data alongside the Museum’s professional scientific staff.

#### *TAS2R38* SNP analysis

DNA was extracted from Epicentre buccal swabs using the Maxwell 16 Buccal Swab LEV DNA Purification Kit and the Promega Maxwell. TAS2R38 was amplified using PCR primers (Forward ACCAATGCCTTCGTTTTCTTGGTGA, Reverse TCACAGCTCTCCTCAACTTGGCA, Invitrogen) and sequenced using the forward primer (High Throughput Genomics Center, Seattle, WA, USA, www.htseq.org). Sequences were analyzed using the program Geneious^2^ to determine the amino acid sequence resulting from the first two SNPs of the gene at nucleotide position 145 and nucleotide position 785 (NCBI Accession AY258598). Only individuals with sequencing data reflective of the three major diplotypes for TAS2R38 (PAV/PAV, AVI/AVI, PAV/AVI) were included in further analysis.

We have a three step process in place for our genetic analysis to prevent inaccurate recording of variations to the gene TAS2R38. Step 1, staff scientists uploaded all sequences into the software program Geneious. The sequences were then aligned to the TAS2R38 reference sequence (AY258598) using the program option “Align, Map to Reference.” Following alignment, staff used the “Find Variations/SNPs” option to highlight the variations in the aligned sequences, and “Find Heterozygotes” to highlight heterozygotes at each variation. Step 2, once this preparation was performed by staff scientists, a small number of citizen scientists trained in chromatograph analysis worked in teams of two to record the diplotype for each sample. Citizen scientists only recorded samples where the chromatograph matched the computer program reading at that nucleotide position, and only if it showed one of the three main diplotypes (e.g., for SNP at position 145: G/G, C/C or G/C). Step 3, any samples that showed discrepancy from the chromatograph to the computer assignment, or showed any other nucleotide other than the two known variations were flagged and analyzed by staff scientists.

#### Bitter taste stimuli and sensitivity

Filter disks were impregnated with a solution of 0.453 M PROP for the taste test (Zhao et al., 2003; Khataan et al., 2009). Participants in the study were first trained to use the general Labeled Magnitude Scale (gLMS; Green et al., 1996; Bartoshuk et al., 2004; Hayes et al., 2013a), and then used the scale to rate the bitterness intensity of the impregnated filter disk. The rating was then converted to a score in millimeters for use in statistical analysis. The base-10 logarithm of the PROP intensity score plus one was used in the subsequent analysis as the gLMS typically generates log-normal data; this transformation reduces skew and results in more normally distributed residuals.

#### Fungiform papillae analysis

Participants’ tongues were temporarily stained blue using ESCO Foods liquid color (deep blue shade), diluted 1:10 with deionized water. Participants steadied their head by placing their chin on fisted hands, with their elbows on the bench. They held a popsicle stick with their unique visitor identification number just to the side of their mouth. A paper disk with a 1 cm circular cut out was placed to the left of the center line of the tongue at the apical tip (Shahbake et al., 2005) and a digital photograph was taken of the lower half of their face to maintain confidentiality. It is of note that we did not flatten the tongue. During photo comparisons, we found that tongues flattened under glass or saran wrap often had a glare and distorted image that the neutral tongue did not. While the glare may have been due partially to the amount of light in our lab and the materials we selected for flattening, we ultimately chose the neutral tongue to prevent the distortion of the papillae. We found distortion to both increase the diameter of the papillae thus obscuring the distinction between fungiform and filiform papillae and prevent one from determining the elevation differences on the tongue. Both of these consequences would have made it difficult to identify the FP density.

**FIGURE 1:**
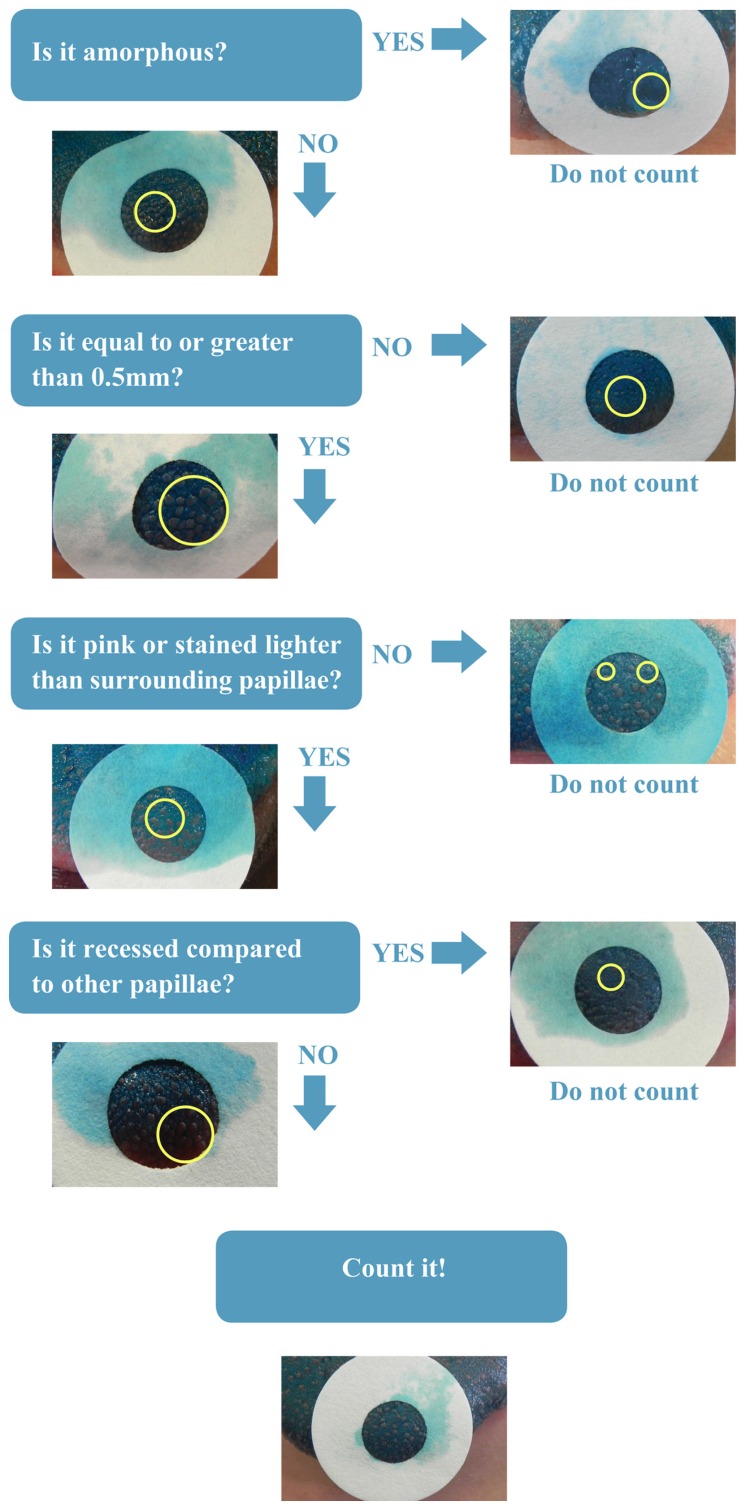
**Denver papillae protocol (DPP) dichotomous key.** Prioritized flow chart of fungiform papillae characteristics.

All papillae counts were completed using the free software ImageJ hosted by the National Institutes of Health^3^. The 1cm area was analyzed by counters who were blind to any other data from that participant. The counters were trained to follow a novel protocol, the Denver Papillae Protocol (or simply DPP), to reduce variance and increase accuracy. DPP is a dichotomous key with clear and distinct characteristics of FP that were selected based on previous literature, but for the first time pooled and prioritized for a more complete and objective method (see **Figure 1**).

Twenty-six counters scored 15 photographs using the standard methodology in the field (Miller and Reedy, 1990a). The counters were then trained on the DPP method and blinded to the goal of the training and to the photo IDs, scored the photographs once more. A mixed linear model was fit with a fixed training effect, a random effect due to counter, and an interaction effect of training on counter, Yijk = μ + τi + ρj + (τρ)ij + εijk (where i = 1,2, j = 1,…, 26 and k = 1,…, 15). The effect of training was significant (*p* = 0.0007). This model demonstrates that 5.2% of the variability in the score was due to training, 25.9% due to counter, and the remaining due to random error. Finally variance due to individual counter within DPP was assessed on 11 reviewers who blindly scored 30 images (24 distinct images, and three repeated twice). Variance decreased significantly from the distinct images (median variance = 140.5) compared to that of the repeated images (median variance = 16.5) demonstrating the ability of the key and training to ensure accurate and repeatable counts.

We used the following processes to ensure FP counts analyzed by our team of citizen scientists are usable. We had a total of 1005 photographs that were counted as part of the full study. We used a simple random sample to verify 10% of the photos (*n* = 100) and asked the question: what proportion of citizen science counts is within 10% of professional scientist’s counts? Defining X = counts that fell within 10% and *n* = the sample number, then the estimated proportion of valid counts is 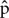 = X/n. We then calculated the 95% confidence interval using the formula $\overset{\wedge }{p}\pm 1.96\sqrt{\frac{\overset{\wedge }{p}\left(1-\overset{\wedge }{p}\right)}{n}}$.

We report a proportion of 0.81 with 95% Confidence Interval of 0.733≤ p≤ 0.887. In addition, after calculating the difference in counts between professional and citizen scientists on the random sample, we find the following numerical summary: the minimum difference between a citizen science counted photograph and a professional scientist counted photograph is 0, the maximum difference observed is 23, the interquartile range is 3.5, the median difference is 3, and the mean difference is 3.77. From these quality control results, we feel confident that the full data set and the statistics derived from citizen science analyzed samples are valid and supported.

## RESULTS

### DOMINANT HAPLOTYPE CARRIERS RATE PROP INTENSITY AS SIGNIFICANTLY HIGHER THAN HOMOZYGOUS RECESSIVE INDIVIDUALS AND FP IS INDEPENDENT OF TAS2R38 DIPLOTYPE

**FIGURE 2:**
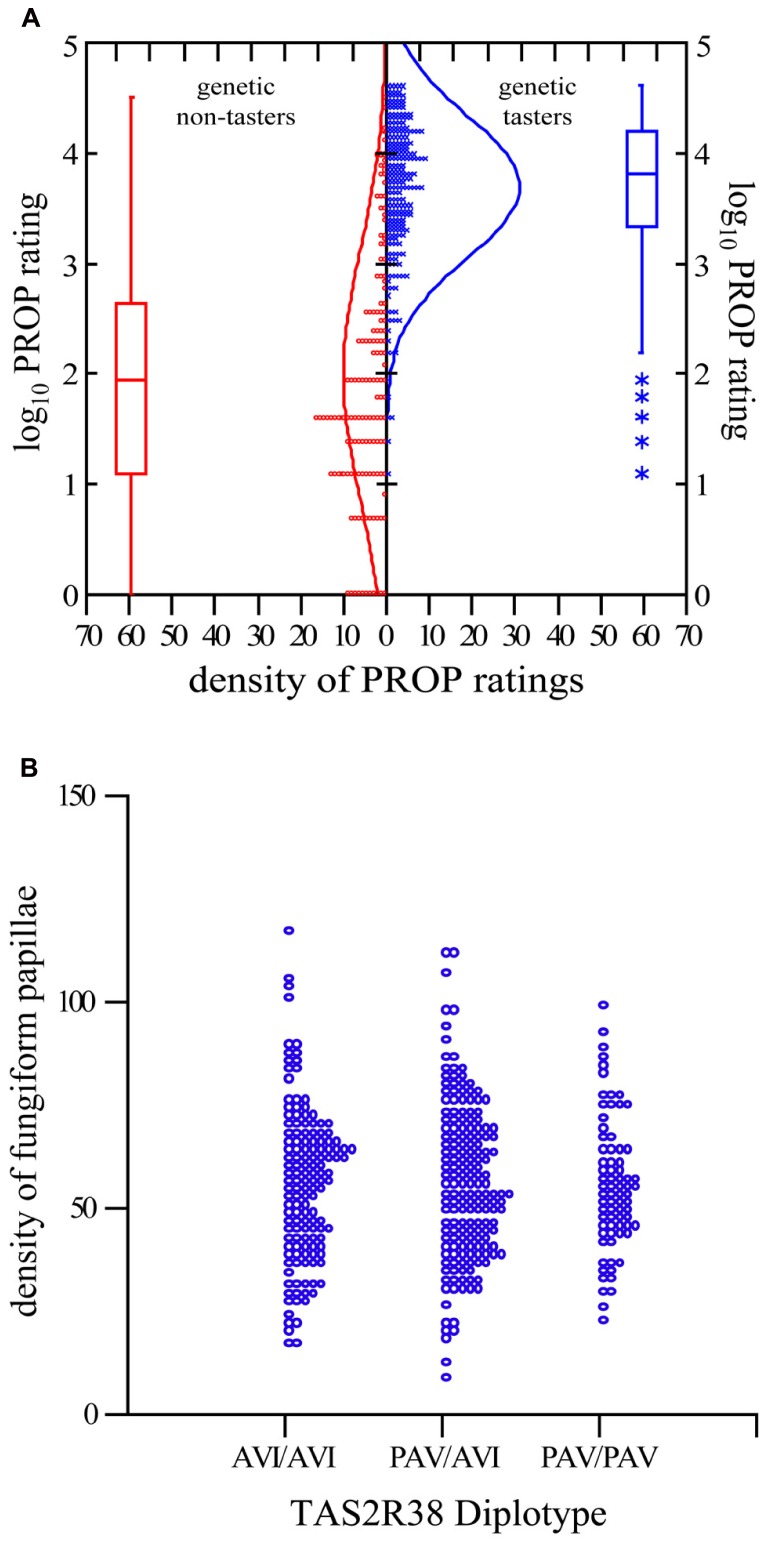
***TAS2R38* diplotype correlates to PROP intensity rating, and not FP. (A)** Two-sample *t*-test comparing the logarithm base 10 of PROP for the genetic non-taster (AVI/AVI) diplotype and genetic taster diplotypes (PAV/AVI and PAV/PAV) *p* <0.0001. Each o/x representing one person. *Indicates outliers. **(B)** Dot density comparing the FP density for the three major diplotypes. Analysis of variance to compare means reports *p* = 0.947. Each *o* represents one person.

To establish the quality control of our community lab we confirmed two key findings from previous studies. First, we used a Student’s *t*-test to confirm that our genetic analysis of the gene *TAS2R38* performed by citizen scientists resulted in previously reported diplotypes, and further confirmed that dominant haplotype carriers (PAV/AVI and PAV/PAV) rated PROP intensity as significantly higher than their homozygous recessive counterparts (AVI/AVI; *p* <0.0001; see **Figure 2A**). Second, using analysis of variance, we then compared the number of FP across *TAS2R38* diplotypes and verified prior reports (Hayes et al., 2008; Fischer et al., 2013) that the number of FP does not differ by diplotype (*p* = 0.947; see **Figure 2B**).

### AGE, SEX, AND DIPLOTYPE PREDICT TASTE SENSITIVITY TO PROP, AND FP DOES NOT

**FIGURE 3:**
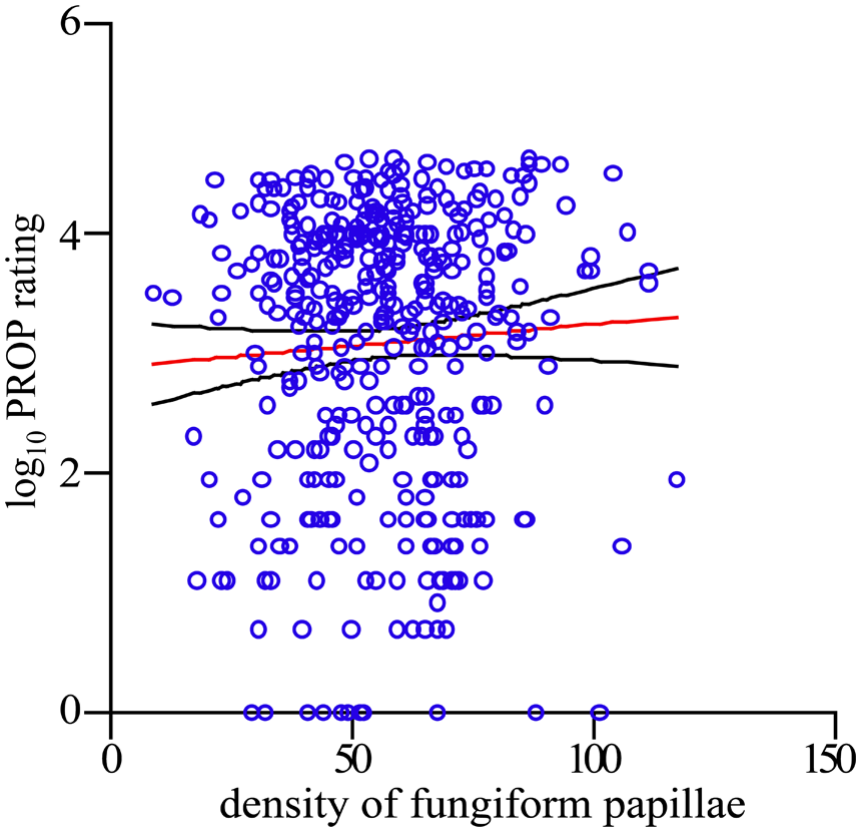
**FP density is not predictive of PROP intensity rating.** Scatterplot of FP per square centimeter and the logarithm base 10 of PROP. Each *o* represents one person. Red line indicates the regression of logarithm base 10 of PROP on FP, and the black lines indicate the upper and lower limits of the 95% confidence interval.

Having established scientific integrity of our crowdsourcing science model through reproducibility, we now were ready to address the unresolved question: does the density of FP on one’s tongue predict the perception of bitterness of the chemical tastant PROP in the phenomenon known as supertasting? First, we employed a simple linear regression model across the entire data set, regressing logged PROP ratings on the predictor FP. We failed to find any evidence that FP associates with PROP response [R-sq = 0.003, *F*(1,392) = 1.23, *p* = 0.267; see **Figure 3**]. Second, we find that FP is not predictive of taste response within each diplotype group, as measured by the logged PROP ratings [linear regression: AVI/AVI R-sq = 0.007, *F*(1,140) = 1.03, *p* = 0.311; PAV/AVI R-sq = 0.004, *F*(1,175) = 0.66, *p* = 0.418; PAV/PAV R-sq = 0.008, *F*(1,73) = 0.59, *p* = 0.447]. However, other factors have been reported to play a role in the ability to taste PROP that may have contributed to this null result. These factors include: sex and age in addition to *TAS2R38* diplotype (Bartoshuk et al., 1994; Kim et al., 2003; Hayes et al., 2008; Tepper et al., 2008; Khataan et al., 2009; Mennella et al., 2010). With this in mind, we used multiple linear regression of the logged PROP ratings on the predictor FP, controlling for the effects of age, sex, and diplotype. This model explains a significant proportion of variance in PROP [R-sq = 0.514, *F*(5,388) = 81.93, *p* <0.0001]; however, the success of this final model to predict taste intensity is only due to the factors age, sex, and diplotype and not due to the inclusion of FP (see **Table 1**).

**Table 1:** FP density is not predictive of PROP intensity rating.

Significance of factors			
Predictor		*b*	*P* value
Diplotype	PAV/AVI	0.7221	<0.0001
	PAV/PAV	0.7818	<0.0001
Sex (male)		-0.1190	=0.002
Age		-0.0038	=0.001
FP		0.0010	=0.306

*Significance of individual predictors using a multiple linear regression model of the logged PROP ratings.*

### FP DOES NOT DIFFER ACROSS TASTE SUBGROUPS NO MATTER THE DIVISION METHOD

This result made us reassess how to approach our original question of the role of FP in PROP taste sensitivity and supertasting. Perhaps the positive relationship only exists when one specifically compares FP between subgroups representing different taste sensitivities like the aforementioned population of supertasters. Therefore, under this hypothesis, groups that contain people with low sensitivity to PROP should exhibit less FP than groups that are composed of people with high PROP sensitivity. Previous reports demonstrate that three populations can be separated out when taste ratings of PROP are plotted (Bartoshuk et al., 1994; Hayes and Pickering, 2012). These three unique PROP taster status groups are typically referred to as non-tasters, medium tasters, and supertasters. Along this line of reasoning, it has been suggested that FP density differs between these three subgroups (Bartoshuk et al., 1994). Differences in FP density are thought to affect more than just PROP response; these differences have been used to explain increased intensity of other tastes and oral sensations. The reasoning follows: if more receptors are stimulated, perceived intensity also increases (Smith, 1971). This implies that, for a fixed area, individuals with more fungiform papillae should report greater taste intensities. Indeed, historically, this has been the case (Miller and Reedy, 1990b; Delwiche et al., 2001). Fungiform papillae are innervated by both taste (cranial nerve VII) and touch nerves (cranial nerve V); it is therefore hypothesized that if higher FP density causes increased PROP response, then it might also explain why individuals who report high sensitivity to PROP bitterness also report greater capsaicin burn (Karrer and Bartoshuk, 1991) and wine astringency (Pickering and Robert, 2006). Despite this, there is disagreement on the validity of this hypothesis, as recently published data from the Beaver Dam Offspring Study reports an inability to replicate the result that FP densities differ between taster status groups (Fischer et al., 2013).

**FIGURE 4:**
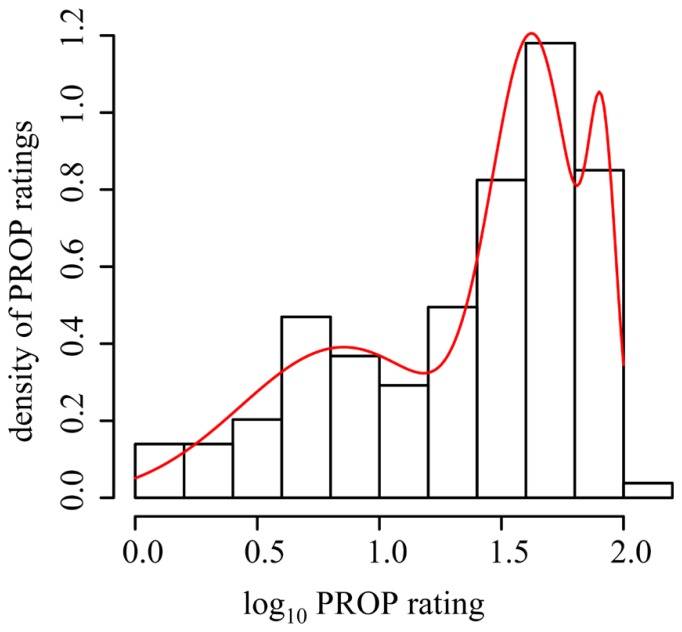
**Division of taster status subgroups using a mixture model of three normal distributions.** Frequency histogram of logarithm base 10 of PROP overlaid with the mixture density overlaid in red. Data statistically divided into the three groups, with group membership assigned via their posterior probability.

Because this recent report and our present data suggest FP is not related to PROP intensity, the Museum specifically wished to test if FP density differed between categorized taster status subgroups. We selected four methods for determining subgroup assignment that would be used for further analysis. The first method was based solely on the distributions observed in our data set. We categorized participants based on a mixture model of three normal distributions of their logged PROP ratings (McLachlan and Peel, 2000). The subgroups were then divided with membership assigned via posterior probability (PROP values for group 1 from 0 to 19, group 2 from 20 to 71 and group 3 from 72 to 100; see **Figure 4**). For the second method we divided the data based on the logical division of a group into tertiles (Gelman and Park, 2008), which led to grouping based on the following ratings: group T1 from 0 to 15, T2 from 16 to 45, and T3 from 46 to 100. The third method we used was *a priori* cutoffs for non-tasters (NT from 0 to 15), medium tasters (MT from 16 to 66), and supertasters (ST from 67 to 100; Zhao et al., 2003). The final method we employed was quartiles which were originally based on assumed Mendelian genetics of the *TAS2R38* gene (Bartoshuk et al., 1994). Following this methodology, the lower quartile (ratings 0–9) is symbolic of the homozygous recessive diplotype, the heterozygous diplotype represents the middle two quartiles (ratings 10–53) and finally, the upper quartile (ratings 54–100) is the homozygous dominant diplotype. The final two methods were specifically selected as they have been previously reported to show a positive relationship between FP and PROP intensity ratings. Using analysis of variance and Tukey’s honestly significant difference test, we then compared the number of FP across each subgroup within each taster grouping method (see **Table 2**). This analysis shows that regardless of the classification method employed to divide the PROP rating data into taster status subgroups, there is no evidence that FP density differs across the subgroups, and therefore does not support the assertion used by scientists and the media alike that highly sensitive tasters (supertasters) bear a higher amount of papillae than less sensitive individuals.

**Table 2:** FP density does not differ across taster status groups.

Division method	Subgroup comparison		*P* value
Mixture model	Group 1	Group 2	0.810
	Group 1	Group 3	0.137
	Group 2	Group 3	0.267
Tertiles	T1	T2	0.899
	T1	T3	0.651
	T2	T3	0.896
*A priori*	NT	MT	0.926
	NT	ST	0.351
	MT	ST	0.466
Quartiles	Lower	Middle two	0.737
	Lower	Upper	0.414
	Middle two	Upper	0.750

*Pairwise comparison using Tukey’s honestly significant difference test shows no difference in FP across subgroups within each division method.
*

## DISCUSSION

Using an advanced model of citizen science in a community-based lab setting, the Genetics of Taste Lab at the Denver Museum of Nature & Science has collected and analyzed population data to assess the putative role of FP density in PROP taste intensity and in the phenomenon known as supertasting. First, our genetic analysis reiterated the well-established relationship between *TAS2R38* diplotypes and PROP intensity, and the lack of a relationship between FP density and *TAS2R38* diplotype, and served to establish the scientific credibility of our community lab. Second, our population data provides no evidence to substantiate prior reports that FP is predictive of PROP intensity rating and that it contributes to supertasting. Based on taste rating plotting, it is clear that there are individuals who are more highly sensitive to PROP, and we suggest further investigation is needed to determine the influence of FP density and enervation on the sensitivity to all taste qualities and oral somatosensation. As taste genetics continues to capture the interest of the media and the general public, and is the foundation for the growing field of personalized nutrition (e.g., Hayes et al., 2013b), these data have large implications for moving beyond the term supertaster to defining hypergeusia and its relationship to food choices (Lim et al., 2008; Hayes and Keast, 2011). Finally, this study demonstrates the capability of crowdsourcing and citizen science models to not only address the shortcomings of small sample size and the labor intensive population data preparation and analysis in behavioral studies, but ultimately for these models to soundly conduct and contribute to scientific research. We hope that this work serves as motivation for more scientists to incorporate citizen science into their research designs to better engage and develop a sustainable dialog with the general public.

## Conflict of Interest Statement

The authors declare that the research was conducted in the absence of any commercial or financial relationships that could be construed as a potential conflict of interest.

## Footnotes

1 http://phrp.nihtraining.com

2 http://www.geneious.com

3 http://rsb.info.nih.gov/ij
